# The Importance of Genetic Background and Neurotransmission in the Pathogenesis of the Co-Occurrence of Sleep Bruxism and Sleep-Disordered Breathing—Review of a New Perspective

**DOI:** 10.3390/jcm13237091

**Published:** 2024-11-23

**Authors:** Joanna Smardz, Andrej Jenca, Sylwia Orzeszek

**Affiliations:** 1 Department of Experimental Dentistry, Wroclaw Medical University, 50-425 Wroclaw, Poland; sylwia.orzeszek@umw.edu.pl; 2 Clinic of Stomatology and Maxillofacial Surgery, Faculty of Medicine, University Pavol Josef Safarik and Akademia Kosice, 041 90 Kosice, Slovakia; andrej.jenca1@upjs.sk

**Keywords:** sleep bruxism, sleep-disordered breathing, obstructive sleep apnea, neurotransmission, genetics, dopamine, serotonin, GABA

## Abstract

Sleep bruxism (SB) and sleep-disordered breathing (SDB) are two prevalent conditions that significantly impact overall health. Studies suggest that up to 49.7% of individuals with SDB also exhibit symptoms of SB. This review aims to provide a comprehensive analysis of the role of genetic background and neurotransmission in the pathogenesis of the co-occurrence of SB and SDB. It seeks to synthesize current knowledge, highlight gaps in the existing literature, and propose a new perspective that integrates genetic and neurobiological factors. This review shows that both SB and SDB may be influenced by a combination of genetic, neurochemical, and environmental factors that contribute to their shared pathophysiology. The key neurotransmitters—dopamine, serotonin, and GABA—may play a significant role in their co-occurrence by regulating motor activity, sleep architecture, and respiratory control. Understanding genetic and neurochemical mechanisms may allow for more precise diagnostic tools and more personalized treatment approaches regarding SB and SDB. Clinically, there is a need for interdisciplinary collaboration between sleep specialists, dentists, neurologists, and geneticists. There is also a need to conduct large-scale genetic studies, coupled with neuroimaging and neurophysiological research, uncovering additional insights into the shared mechanisms of SB and SDB.

## 1. Introduction

Sleep bruxism (SB) and sleep-disordered breathing (SDB) are two prevalent conditions that significantly impact sleep quality and overall health. In accordance with the consensus from 2018, SB is defined as a masticatory muscle activity during sleep that is characterized as rhythmic (phasic) or non-rhythmic (tonic) and is not a movement disorder or a sleep disorder in otherwise healthy individuals [1]. It is commonly associated with symptoms such as masticatory muscle pain, headaches, and tooth wear [1,2,3,4]. However, it should be noted that in otherwise healthy individuals, bruxism should not be considered as a disorder, but rather as a behavior that can be a risk (and/or protective) factor for certain clinical consequences [1]. Conversely, SDB encompasses a range of conditions, including obstructive sleep apnea (OSA), characterized by repeated episodes of partial or complete upper airway obstruction during sleep, leading to intermittent hypoxia, sleep fragmentation, and cardiovascular stress [5].

The global prevalence of SB is 21%, and the occurrence based on polysomnography is estimated at 43% [6]. Approximately 1 billion of the world’s population of 7.3 billion people, between the ages of 30 and 69 years, are estimated to have the most common type of sleep-disordered breathing, OSA [7]. The co-occurrence of SB and SDB is increasingly recognized in clinical settings, with studies suggesting that up to 49.7% of individuals with SDB also exhibit symptoms of SB [8]. The co-occurrence of SB and SDB complicates the clinical management of affected individuals, often requiring multidisciplinary approaches involving dentists, sleep specialists, and neurologists. Furthermore, this overlap poses challenges in diagnosis and treatment, as symptoms can be mistakenly attributed to one condition without recognizing the contribution of the other [8,9].

Historically, research has treated SB and SDB as distinct entities with separate pathophysiological mechanisms. SB was primarily associated with psychological stress, dental occlusion, and peripheral sensory factors, while SDB was linked to anatomical abnormalities, obesity, and neuromuscular control of the airway [10,11]. Early studies explored the role of micro-arousals from SDB events in triggering SB episodes, but the causative relationship remained ambiguous [12]. The more recent literature has shifted towards exploring common pathophysiological pathways, such as autonomic nervous system dysregulation and shared environmental risk factors, yet a comprehensive understanding of the interaction between these conditions remains limited [13,14].

The emerging interest in the genetic and neurotransmission aspects of these conditions stems from the recognition that both SB and SDB may share common neurobiological underpinnings. Genetic studies have identified potential links between genes regulating neurotransmission, especially the serotonin pathway, and the predisposition to both conditions. Wieckiewicz et al. reported that 5-hydroxytryptamine receptor 2A (HTR2A) rs2770304 polymorphism might contribute to the association between SB and SDB [15]. Additionally, neurotransmission imbalances affecting sleep regulation, arousal responses, and muscle tone may contribute both to SB and SDB [15,16,17,18,19,20,21]. By focusing on genetic background and neurotransmission pathways, this review aims to uncover novel insights into the shared pathogenesis, which could pave the way for more targeted and effective therapeutic strategies.

This review aims to provide a comprehensive analysis of the role of genetic background and neurotransmission in the pathogenesis of the co-occurrence of SB and SDB. It seeks to synthesize current knowledge, highlight gaps in the existing literature, and propose a new perspective that integrates genetic and neurobiological factors. The scope of this review includes a detailed examination of genetic studies, neurotransmission pathways, and their interaction, with a focus on how these factors may influence the development and manifestation of both conditions. By offering an updated perspective, this review hopes to contribute to a deeper understanding of the complex interplay between genetic predisposition, neurotransmission, and the co-occurrence of SB and SDB, ultimately informing future research and clinical practice.

## 2. Pathophysiology

### 2.1. Pathophysiology of Sleep Bruxism 

Although the exact cause of SB remains unclear, several key factors have been identified in its pathogenesis.

#### 2.1.1. Central Nervous System Dysfunction

SB is believed to be primarily driven by central nervous system processes, with a focus on the brainstem and its regulation of motor activity during sleep [21,22,23]. Studies have shown that SB episodes are often preceded by an arousal from sleep, which activates motor neurons in the brainstem, leading to rhythmic masticatory muscle activity (RMMA). These arousals are usually associated with transient autonomic activity, such as increased heart rate and blood pressure, suggesting a link between autonomic nervous system dysregulation and SB [1,10,21,22,23,24,25,26,27].

#### 2.1.2. Autonomic Nervous System Dysregulation

It is believed that dysregulation of the autonomic nervous system, specifically the sympathetic branch, plays a significant role in SB [26,28,29]. Bruxism episodes are often preceded by shifts in autonomic activity, which may be triggered by cortical arousals. This heightened autonomic activity may increase the likelihood of SB episodes by stimulating the motor neurons involved in jaw muscle activity [26,28,29,30,31,32,33].

#### 2.1.3. Genetic and Neurotransmitter Influences

Genetic predisposition also plays a role in the development of SB. Research has identified potential genetic markers, including polymorphisms in genes that regulate neurotransmitter systems, such as dopamine and serotonin [15,34,35,36]. Severe SB was reported to be associated with lower serotonin levels, but also with no differences in the level of enzymes involved in serotonin synthesis [16,17]. Dopamine dysfunction has been also implicated in the pathophysiology of sleep bruxism due to its role in motor control and arousal regulation [15,22,29,36].

#### 2.1.4. Psychological and Behavioral Factors

While SB is primarily considered a motor condition, psychological factors such as stress, anxiety, and emotional disturbances have been potentially linked to increased bruxism activity [1,29,34,37,38,39,40,41]. These factors may exacerbate arousal responses during sleep, further contributing to the onset of SB episodes [40,41].

#### 2.1.5. Other Risk Factors

As SB seems to be largely influenced by genetic and neurological factors, exogenous factors such as caffeine, nicotine, and alcohol have been also linked to increased bruxism episodes [1,29,42]. These external influences can heighten arousal responses during sleep, further triggering masticatory muscle activity [43,44]. Sleep disorders like OSA and environmental factors, such as noise, can also disrupt sleep and exacerbate bruxism [1,42,44]. In accordance with the 2024 meta-analysis geographical region and ethnological factors may also be considered as new factors in the pathophysiology of SB [6].

### 2.2. Mechanisms Underlying Sleep-Disordered Breathing

SDB encompasses a spectrum of conditions characterized by abnormal breathing patterns during sleep. OSA is the most common form, in which repetitive episodes of partial or complete obstruction of the upper airway occur, leading to reductions in airflow (hypopnea) or complete cessation of airflow (apnea) [5]. Below we present the primary pathophysiological mechanisms underlying OSA.

#### 2.2.1. Upper Airway Obstruction

OSA is caused by intermittent collapse of the upper airway during sleep, particularly in the oropharyngeal region. This collapse is often due to anatomical factors, such as a narrow airway, enlarged tonsils, or excessive tissue in the throat. In some cases, abnormal airway tone or muscle activity in the pharyngeal dilator muscles also contribute to airway obstruction [45,46].

#### 2.2.2. Arousal-Induced Breathing Resumption

During an OSA event, blood oxygen levels drop, leading to hypoxia and hypercapnia, which is increased levels of carbon dioxide [45,46]. This may trigger an arousal response in the brain, which reactivates the muscles that open the airway, allowing for breathing resumption. These frequent arousals disrupt the continuity of sleep, resulting in poor sleep quality and fragmentation, which are hallmark features of OSA [45,46,47].

#### 2.2.3. Autonomic and Cardiovascular Implications

Repeated episodes of apnea and hypopnea lead to intermittent hypoxia, which stimulates the sympathetic nervous system, resulting in surges in blood pressure and heart rate during sleep. This autonomic activation may be associated with long-term cardiovascular consequences, including hypertension, arrhythmias, and an increased risk of heart disease [45,48,49].

#### 2.2.4. Risk Factors for Obstructive Sleep Apnea 

The risk factors for OSA include obesity, male gender, advanced age, and anatomical abnormalities. Excess body weight is particularly important, as fat deposits around the upper airway can contribute to airway narrowing and collapse [50]. Additionally, conditions such as nasal obstruction, alcohol use, and sedative medications can worsen OSA by relaxing the muscles that keep the airway open [45,51]. It is reported that the prevalence of OSA varies depending on geographical region [7].

### 2.3. Potentially Common Pathways and Physiological Overlap of Sleep Bruxism and Sleep-Disordered Breathing 

The co-occurrence of SB and SDB, particularly OSA, is increasingly recognized in clinical research. Several common physiological pathways may explain this overlap.

#### 2.3.1. Autonomic Nervous System Dysregulation

Both SB and OSA involve dysregulation of the autonomic nervous system, particularly in response to arousals during sleep [1,10,21,22,23,24,25,26,27,28,29,30,31,32,33,45,46,47,48,49]. In OSA, episodes of airway obstruction trigger sympathetic activation, leading to a spike in autonomic activity [45,48,49]. Similarly, SB episodes are associated with transient surges in autonomic function, suggesting that heightened sympathetic activity may be a shared mechanism between these two conditions. The micro-arousals caused by apneic events in OSA may serve as triggers for SB episodes, further linking them [52,53,54].

#### 2.3.2. Arousal Mechanisms

The arousal response plays a crucial role in both conditions. In OSA, arousals from sleep are necessary to resume breathing after airway obstruction, serving as protective factor. Furthermore, SB episodes were hypothesized to be a protective factor for OSA as mandibular movements can influence restoring airflow through the respiratory tract [1,25,29]. These arousals are often accompanied by an increase in RMMA, potentially triggering bruxism episodes. Studies have shown that a significant proportion of SB events occur shortly after apneic episodes, supporting the hypothesis that OSA-induced arousals may precipitate SB [26,52,53,54,55].

#### 2.3.3. Shared Risk Factors

Both SB and OSA share several common risk factors, including obesity, alcohol consumption, and smoking. These factors can exacerbate both conditions by increasing the likelihood of airway obstruction and intensifying autonomic activity during sleep [56,57]. Additionally, stress and anxiety, which are known to increase the frequency of SB, may also contribute to SDB, highlighting the complex interplay between psychological and physiological factors [58,59]. There is also the possibility of the occurrence of shared geographical and/or ethnological factors [6,7].

#### 2.3.4. Genetic and Neurotransmission Susceptibility

Emerging research suggests that there may be genetic factors that predispose individuals to both SB and OSA [15]. Genes involved in the regulation of neurotransmitters such as dopamine and serotonin, which influence arousal and motor control, may contribute to the co-occurrence of these conditions; as an example, serotonin was reported to be potentially involved both in SB and SDB [16,17,18]. While the genetic basis of this overlap is still being explored, early findings indicate that shared genetic predispositions may play a role in the pathophysiology of both entities [15,16,17,18,60].

**Figure 1:**
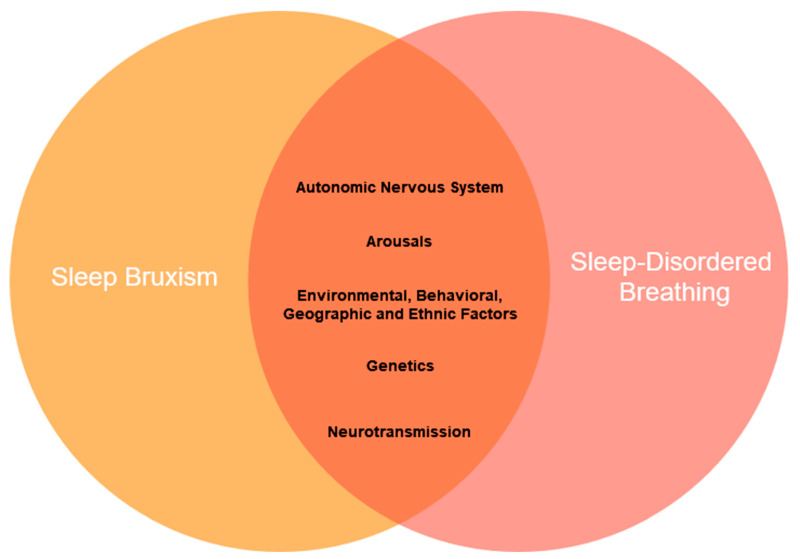
Potentially common pathways and physiological overlap of sleep bruxism (SB) and sleep-disordered breathing (SDB).

Figure 1 presents common pathways and physiological overlap of SB and SDB.

## 3. Genetic Factors

### 3.1. Genetic Factors Implicated in Sleep Bruxism 

In the last 15 years, increasing evidence suggests that genetic predisposition may play a significant role in the development of SB. Several studies have explored the hereditary nature of bruxism, indicating a familial tendency for the condition [1,19,61,62]. Twin studies have been particularly useful in establishing the genetic basis of bruxism, showing that monozygotic twins have a higher concordance rate for bruxism compared to dizygotic twins [61,62]. This suggests that genetic factors may contribute to the likelihood of developing the condition.

One of the primary areas of genetic research on SB focuses on the role of neurotransmitter regulation, particularly genes that influence the dopaminergic and serotonergic pathways. Variants in the DRD1, DRD2, and DRD3 genes, which encode the dopamine D1, D2, and D3 receptors, have been implicated in bruxism. This is consistent with the understanding that dopamine plays a critical role in motor control and arousal regulation, both of which are believed to be involved in the pathogenesis of SB [17,36,63,64]. Additionally, genes involved in serotonin regulation, such as HTR2A, have been linked to SB, further supporting the hypothesis that neurochemical imbalances in the brain may contribute to it [15,35,36,65,66]. What is more, associations of bruxism and metalloproteinase 9 (MMP9) and cathecol-o-methyltransferase (COMT) genes in adults and alpha-actinin-3 (ACTN3) gene in children were also reported [67,68].

### 3.2. Genetic Determinants of Sleep-Disordered Breathing 

SDB—particularly OSA—may be also influenced by genetic factors. Studies have shown that OSA tends to cluster in families, suggesting a heritable component [69]. Studies have identified several genetic loci associated with OSA, particularly those involved in craniofacial structure, obesity, and neuromuscular control of the airway [69,70,71].

For example, polymorphisms in genes such as PTEN and FOXC2, which are involved in craniofacial development, have been linked to a higher risk of airway obstruction, a key feature of OSA [72,73]. Additionally, genes related to adiposity, such as FTO and LEP (leptin), have been associated with increased body mass index and a higher likelihood of developing OSA. Since obesity is a major risk factor for OSA, these genetic findings highlight the importance of metabolic pathways in the pathophysiology of SDB [74].

Further, genes that regulate neuromuscular control of the airway, such as PHOX2B, which is involved in the autonomic nervous system and control breathing, may also be implicated in OSA. These genetic factors contribute to a weakened ability to maintain airway patency during sleep, leading to repeated episodes of SDB [75,76].

### 3.3. Shared Genetic Risk Factors and Their Influence on Co-Occurrence of Sleep Bruxism and Sleep-Disordered Breathing 

The co-occurrence of SB and SBD could be partly explained by overlapping genetic influences on neurochemical systems, craniofacial structure, and autonomic nervous system regulation. Unfortunately, there are not many studies on the genetic basis of the co-occurrence of SB and SDB [15,77].

One potential shared mechanism involves the dopaminergic system. Variants in the DRD2 gene, as mentioned earlier, have been associated with bruxism and also with sleep disorders, including restless legs syndrome and periodic limb movement disorder, both of which potentially share some neurophysiological similarities with OSA. The involvement of dopamine in motor control and arousal regulation suggests that disturbances in dopamine could contribute to both conditions, particularly through mechanisms that involve arousal-induced muscle activity [29,30,36,64,78].

A second potential mechanism involves the serotonergic system. Wieckiewicz et al. reported that HTR2A rs2770304 polymorphism might contribute to the association between SB and OSA [15].

In summary, genetic factors seem to play a significant role in both SB and SDB, with only potentially shared genetic pathways contributing to their co-occurrence. While the exact mechanisms remain under investigation, familial clustering, heritability studies, and the identification of specific genetic markers provide valuable insights into the complex etiology of these conditions. Further research is needed to explore the genetic overlap between SB and SDB and understand how genetic and environmental factors interact to influence the development and progression of these conditions.

**Figure 2:**
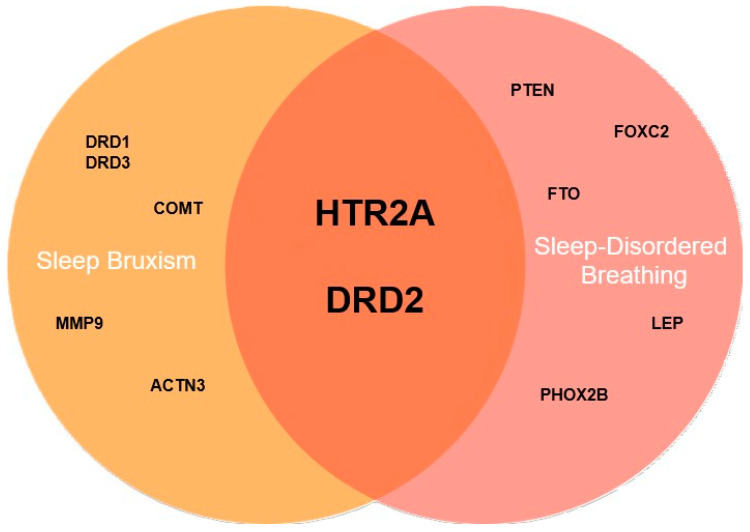
Potentially shared genetic risk factors between sleep bruxism (SB) and sleep-disordered breathing (SDB). Legend: ACTN3—gene encoding alpha-actinin-3, COMT—gene encoding catechol-O-methyltransferase, DRD1—gene encoding dopamine receptor D1, DRD2—gene encoding dopamine receptor D2, DRD3—gene encoding dopamine receptor D3, FOXC2—gene encoding forkhead box protein C2, FTO—gene encoding fat mass and obesity-associated protein, HTR2A—gene encoding 5-hydroxytryptamine receptor 2A, LEP—gene encoding leptin, PHOX2B—gene encoding paired-like homeobox 2B.

Figure 2 presents potentially shared genetic risk factors between SB and SDB.

## 4. Neurotransmission and Neuromodulation

### 4.1. Overview of Neurotransmitters Involved in Sleep Regulation, Muscle Activity, and Respiratory Control

Neurotransmitters play a crucial role in regulating the different stages of sleep, motor activity, and respiratory control, all of which are involved in both SB and SDB. Sleep is governed by a delicate balance between excitatory and inhibitory neurotransmitters that modulate arousal, muscle tone, and respiratory patterns. Key neurotransmitters involved in these processes include dopamine, serotonin, and gamma-aminobutyric acid (GABA) [79,80,81,82].

Serotonin is a neurotransmitter in the central nervous system derived from tryptophan [83]. Most serotonin is synthesized in the gastrointestinal tract, while only a small percentage, approximately 5%, is produced in the central nervous system, especially in the raphe nuclei located in the brainstem [84,85]. Approximately 90% of the total human body’s serotonin is stored in the enterochromaffin cells in the gastrointestinal tract, 8% is stored in blood platelets, and 1–2% is stored in the central nervous system [83,84,85]. The serotonin system functions through a complex receptor family, including seven subtypes, which mediate both excitatory and inhibitory signals. Serotonin is reported to be mainly involved in cognition, attention, emotion, pain, sleep, and arousal [83,84,85].

Dopamine is a chemical compound from the group of biogenic catecholamines. It belongs to the same group of substances as adrenaline and norepinephrine. It is synthesized from tyrosine. The biosynthesis of catecholamines occurs in adrenergic neurons and in the chromaffin cells of the adrenal medulla. Dopaminergic neurons are primarily localized in the substantia nigra pars compacta and the ventral tegmental area, projecting to the striatum, frontal cortex, and limbic structures [86]. Dopamine affects many key processes in the human body, acting as both a neurotransmitter and a hormone. In the nervous system, it is responsible for regulating. Dopamine is integral to the regulation of motor control, reward processing, emotions, and executive functions. As a hormone, dopamine influences the cardiovascular system [86,87].

GABA is the primary inhibitory neurotransmitter in the central nervous system, synthesized from glutamate. GABAergic neurons are distributed ubiquitously throughout the brain, with dense networks in the cortex, hippocampus, and cerebellum [88,89]. GABA exerts its effects via GABA_A and GABA_B receptors, which mediate fast synaptic inhibition and slow modulatory inhibition, respectively [89]. The GABA system is critical for maintaining neural circuit stability. It influences mood, sleep, and muscle tone [88,89].

Dopamine and serotonin are particularly important in modulating arousal, motor function, and mood, while GABA acts as the primary inhibitory neurotransmitter in the central nervous system, promoting sleep and suppressing motor activity. The interactions between these neurotransmitters are critical in ensuring smooth transitions between sleep stages, maintaining airway patency during sleep, and regulating muscle tone [79,82,90].

### 4.2. Potential Role of Neurotransmitters in Sleep Bruxism and Sleep-Disordered Breathing 

Considering the available literature on the impact of neurotransmission and neuromodulation on SB and SBD, the following chapter will mostly consist of considerations on the activity patterns of individual substances on the co-occurrence of SB and SBD.

#### 4.2.1. Dopamine

Dopamine is a key modulator of motor control, arousal, and reward pathways [90]. It could potentially play role in both SB and SBD [91,92]. There is a lack of original research indicating the direct influence of dopamine on SB. However, considering the role of dopamine in sleep, its dysfunction may potentially contribute to the abnormal activation of motor pathways during sleep that may be associated with SB [93].

The dopaminergic system may be also implicated in the pathophysiology of SDB, especially in relation to the control of upper airway muscles. Dopamine influences respiratory motor output and has been shown to affect the activity of muscles that maintain airway patency [94]. In individuals with OSA, disruptions in dopamine signaling may potentially impair the ability to maintain adequate airway tone during sleep, contributing to airway collapse [92,93,94,95,96].

#### 4.2.2. Serotonin

Serotonin is involved in regulating both sleep architecture and respiratory function. It plays a critical role in maintaining muscle tone in the upper airway and modulating arousal thresholds during sleep [60,97,98]. In the context of bruxism, reduced serotonin levels were reported to be associated with increased motor activity during sleep [16,17].

In SDB, serotonin’s role is particularly important in maintaining airway stability. Serotonergic neurons in the brainstem help control the muscles responsible for keeping the upper airway open. In OSA, disruptions in serotonergic signaling may reduce airway muscle tone, making the airway more susceptible to collapse during sleep. Additionally, serotonin helps regulate arousal from sleep, and abnormal serotonin levels may contribute to the increased arousals seen in both SB and OSA [26,31,60,97,98]. What is more, the tendency to SDB seems to co-occur with lower blood serotonin and higher tryptophan hydroxylase 1 (involved in serotonin synthesis) levels [18].

#### 4.2.3. Gamma-Aminobutyric Acid 

GABA is the primary inhibitory neurotransmitter in the central nervous system and is essential for promoting restful sleep and inhibiting motor activity [99]. Potentially, in SB, reduced GABAergic inhibition may lead to excessive motor activity, particularly in the muscles of orofacial area [91,100,101].

In SDB, GABA plays a role in regulating the excitability of neurons involved in respiratory control. GABAergic neurons help maintain a balance between excitation and inhibition within respiratory circuits, ensuring that breathing remains stable during sleep. Disruptions in GABAergic signaling may lead to instability in respiratory patterns, contributing to the apneas and hypopneas seen in OSA [102,103].

In conclusion, neurotransmission and neuromodulation may play crucial roles in the pathophysiology of SB and SDB. Imbalances in key neurotransmitters, such as dopamine, serotonin, and GABA, may affect motor control, arousal thresholds, and respiratory stability, providing potential mechanisms for the co-occurrence of these conditions [16,17,18,59,91,92,93,94,95,96,97,98,99,100,101,102,103]. Understanding these neurochemical pathways may potentially offer new insights into the treatment of individuals suffering from both SB and SBD, potentially leading to more targeted and effective therapeutic interventions.

**Figure 3:**
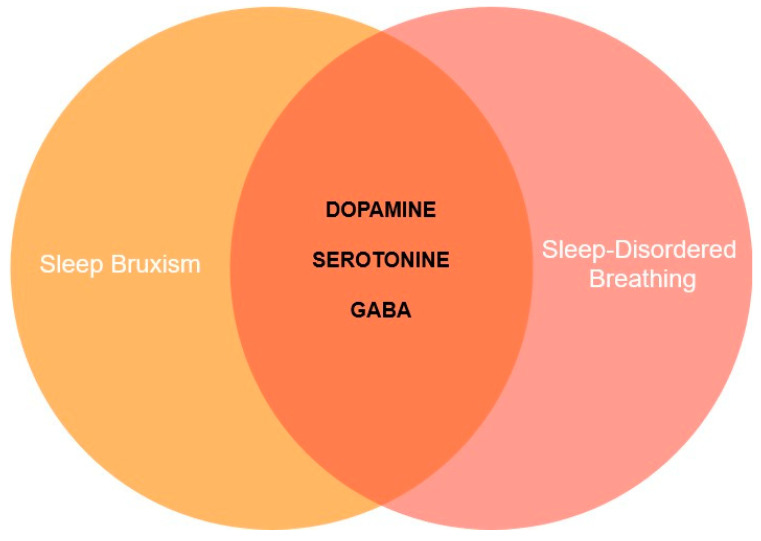
Neurotransmitters potentially involved in sleep bruxism (SB) and sleep-disordered breathing (SDB) co-occurrence. Legend: GABA—gamma-aminobutyric acid.

Figure 3 presents neurotransmitters potentially involved in SB and SDB co-occurrence.

## 5. Clinical Implications and Future Directions

### 5.1. Clinical Implications

The potential integrated pathogenetic model proposes that the co-occurrence of SB and SDB may be the result of complex interactions between genetic predisposition, neurotransmitter imbalances, and autonomic nervous system dysregulation. These factors converge to create an environment in which arousal-driven activation of motor pathways and impaired muscle tone contribute to the development of both SB and SDB [1,10,22,23,24,25,26,27,28,29,30,31,32,33,34,35,36,37,38,39,40,41,42,43,44,45,46,47,48,49,50,51,52,53,54,55,56,57,58,59]. Understanding the genetic and neurochemical mechanisms underlying this co-occurrence can provide valuable insights into the pathophysiology of these conditions and lead to more targeted therapeutic approaches. As research continues to uncover the genetic and neurotransmitter pathways involved, this integrated model will help guide future studies and improve clinical outcomes for individuals with co-occurring SB and SDB.

The growing body of research linking genetic predisposition and neurotransmission dysfunction to the co-occurrence of SB and SDB offers significant implications for clinical management [15,16,17,18,35,36,59,60,61,62,63,64,65,66,67,68,69,70,71,72,73,74,104,105,106]. Recognizing these underlying biological factors can fundamentally change how these conditions are diagnosed and treated, moving beyond symptom management toward a more targeted, individualized approach [107].

By understanding the role of genetic and neurotransmitter imbalances, clinicians can improve the accuracy of diagnosing co-occurring SB and SDB. For example, patients with a known family history of either condition or specific genetic markers associated with dopamine, serotonin, or GABA dysfunction could be flagged for more detailed assessments. This could include using comprehensive sleep studies (polysomnography) or genetic screenings to identify patients at higher risk for both SB and OSA. Identifying these risk factors early could lead to proactive treatment strategies that prevent the progression of symptoms.

Since neurotransmitter imbalances may play a key role in both SB and SDB, pharmacological interventions targeting specific neurotransmitter systems could offer new therapeutic options. Medications that influence dopaminergic, serotonergic, or GABAergic activity may help normalize the neurotransmission imbalances responsible for triggering SB and influencing airway tone in OSA.

Understanding the shared mechanisms between SB and SDB emphasizes the need for a multidisciplinary treatment approach. Patients presenting with SB could benefit from referrals to sleep specialists, while individuals diagnosed with OSA may be screened for SB as part of an integrated care pathway. Collaboration between dentists, sleep specialists, and neurologists can lead to more comprehensive care, with coordinated treatments targeting the root causes of both conditions.

### 5.2. Future Directions

One of the most exciting clinical implications of the research on genetic and neurotransmitter factors is the potential for personalized, precision-based treatment approaches. Rather than using a one-size-fits-all model, clinicians could tailor treatments based on a patient’s specific genetic profile and neurochemical imbalances.

As genetic research continues to advance, clinicians may be able to use genetic profiling to identify patients at risk for co-occurring SB and SDB. For example, patients with certain polymorphisms in genes such as DRD2 and HTR2A could be considered at higher risk for both conditions. These individuals might benefit from earlier interventions, such as lifestyle changes, stress management, or pharmacological treatments, to prevent the onset or progression of SB and OSA [15,29,30,36,64,77,78].

Personalized treatments could also involve the use of neurotransmitter-targeted therapies. For example, patients with dopamine-related genetic predispositions might respond well to dopaminergic drugs, while those with serotonin-related issues could benefit from serotonin precursors [16,17,18,92,93,94,95,96,97,98]. Similarly, patients with GABA-related dysfunction could be treated with GABAergic agents to stabilize motor activity and improve respiratory control during sleep [99,100,101,102,103]

The field of genetic research into SB and SDB is still in its early stages, with much more to be discovered. Future studies could focus on identifying additional genetic markers that contribute to the co-occurrence of these conditions. Large-scale genome-wide association studies could help pinpoint new genetic variants associated with neurotransmitter regulation, muscle activity, and sleep architecture [15,77]. Further research could also explore the epigenetic mechanisms that influence gene expression in response to environmental factors like stress, diet, and lifestyle, leading to a more nuanced understanding of how these conditions develop [108,109].

There is also significant potential for future research into pharmacological interventions targeting the neurotransmitter systems involved in SB and SDB. Clinical trials could investigate the efficacy of drugs that modulate dopamine, serotonin, or GABA activity in reducing the frequency and severity of SB episodes and improving respiratory function in OSA [15,16,17,18,92,93,94,95,96,97,98,99,100,101,102,103]. In particular, combination therapies that address both neurotransmitter imbalances and other risk factors, such as obesity or upper airway dysfunction, may offer the most promising results.

Developing integrative diagnostic models that combine genetic, neurochemical, and clinical data could revolutionize the way SB and SDB are diagnosed and treated. Such models would consider a patient’s genetic predisposition, neurotransmitter imbalances, autonomic regulation, and behavioral risk factors to provide a comprehensive assessment. These models could be used to develop personalized care plans, incorporating both pharmacological and non-pharmacological treatments to optimize patient outcomes. Additionally, these diagnostic models could improve early detection, allowing for preventive measures to be implemented before symptoms worsen.

Advances in sleep technology, such as wearable devices and at-home sleep monitors, could play a role in future research and treatment approaches. These devices can provide real-time data on sleep patterns, muscle activity, and respiratory function, allowing for more detailed assessments of how SB and SDB interact during sleep. Incorporating these technologies into clinical practice could enable more precise monitoring of treatment efficacy and better adaptation of therapy over time [1,110,111].

To better understand the long-term impact of genetic and neurotransmitter factors on the co-occurrence of SB and SDB, longitudinal studies are needed. These studies should track patients over time to assess how genetic predispositions and neurotransmitter imbalances influence the progression of these conditions. Additionally, multicenter trials involving diverse populations could provide insights into how these factors vary across different demographic groups, helping to ensure that personalized treatments are effective for a broad range of patients.

**Figure 4:**
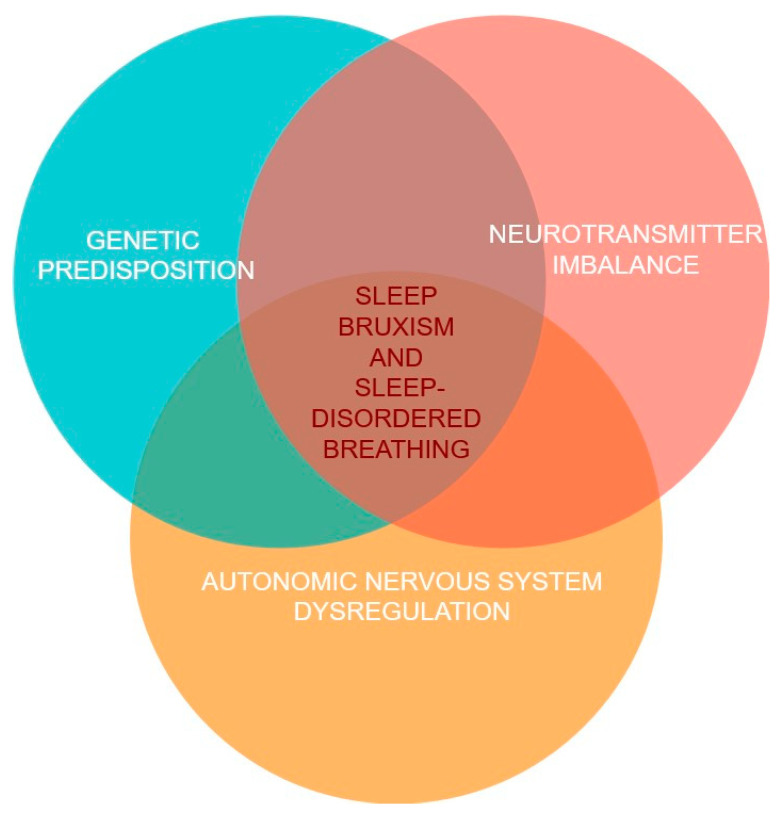
The potential integrated pathogenetic model of sleep bruxism (SB) and sleep-disordered breathing (SDB) co-occurrence.

Figure 4 presents the potential integrated pathogenetic model of SB and SDB co-occurrence.

## 6. Discussion

This review has explored the complex relationship between SB and SDB, particularly OSA, focusing on the role of genetic predisposition and neurotransmission pathways in their co-occurrence. Both conditions may be influenced by a combination of genetic, neurochemical, and environmental factors that contribute to their shared pathophysiology. The research highlights key neurotransmitters—dopamine, serotonin, and GABA—that may play significant roles in regulating motor activity, sleep architecture, and respiratory control, all of which are critical in understanding the overlapping mechanisms of SB and OSA. Genetic studies have provided insights into polymorphisms related to neurotransmitter function, autonomic regulation, and craniofacial development, offering a clearer understanding of how these conditions may develop together.

These findings underscore the importance of considering SB and SDB not as isolated disorders, but as interconnected conditions that share common biological pathways. The co-occurrence of these disorders presents a unique set of challenges for diagnosis and treatment, which can be better addressed by incorporating genetic and neurochemical factors into clinical practice.

The exploration of genetic and neurotransmission perspectives is critical for advancing our understanding of the pathogenesis of SB and SDB. Genetic predispositions, such as polymorphisms in dopamine, serotonin, and GABA-related genes, may play a key role in influencing neurotransmission pathways that regulate sleep, motor control, and respiratory function. These imbalances can lead to heightened motor activity in the jaw muscles during sleep (bruxism) and impaired upper airway muscle tone (OSA), creating a feedback loop that exacerbates both conditions.

Understanding these genetic and neurochemical mechanisms allows for more precise diagnostic tools and more personalized treatment approaches. For example, individuals with specific genetic markers may benefit from targeted therapies that modulate neurotransmitter function, such as dopamine agonists or GABA-enhancing agents. Furthermore, recognizing the role of autonomic dysregulation in both conditions may help refine treatment strategies, focusing on stabilizing arousal thresholds and reducing sympathetic activation during sleep.

This perspective also highlights the need for interdisciplinary collaboration between sleep specialists, dentists, neurologists, and geneticists. By integrating these fields, clinicians can offer more comprehensive care to patients, addressing not only the symptoms of bruxism and OSA but also the underlying neurobiological factors that drive their co-occurrence.

Future research should continue to explore the genetic factors and neurotransmitter pathways involved, with an emphasis on identifying biomarkers that can guide treatment decisions. Large-scale genetic studies, coupled with neuroimaging and neurophysiological research, will be key to uncovering additional insights into the shared mechanisms of SB and OSA. As this field progresses, the integration of genetic screening into routine clinical practice could lead to earlier diagnoses and more targeted interventions, ultimately improving patient outcomes and quality of life.

In conclusion, the genetic and neurotransmitter perspectives on the co-occurrence of SB and SDB provide a fresh and promising framework for understanding these complex conditions. This integrated approach has the potential to revolutionize how we diagnose, treat, and manage these conditions, opening the door to more personalized and effective therapies that address the underlying causes of both SB and OSA. Through continued research and collaboration, we can develop more comprehensive strategies to improve patient care and enhance overall health outcomes.

## 7. Conclusions

- Both SB and SDB may be influenced by a combination of genetic, neurochemical, and environmental factors that contribute to their shared pathophysiology.
- The key neurotransmitters—dopamine, serotonin, and GABA—may play a significant role in SB and SDB co-occurrence by regulating motor activity, sleep architecture, and respiratory control.
- Understanding genetic and neurochemical mechanisms allows for more precise diagnostic tools and more personalized treatment approaches regarding SB and SDB.
- Clinically, there is a need for interdisciplinary collaboration between sleep specialists, dentists, neurologists, and geneticists.
- There is a need to conduct large-scale genetic studies, coupled with neuroimaging and neurophysiological research, uncovering additional insights into the shared mechanisms of SB and SDB.
